# The CADENCE pilot trial – Promoting physical activity in bladder cancer survivors: A protocol paper

**DOI:** 10.1016/j.conctc.2021.100809

**Published:** 2021-06-18

**Authors:** Lee Smith, Anne Carrie, Mark Tully, Yvonne Barnett, Laurie Butler, Claire Gillvray, Rosie Lindsay, Adam Abbs, Mike Trott, Olawale Olanrewaju, Lin Yang, Cristian Petre Ilie

**Affiliations:** a The Cambridge Centre for Sport and Exercise Sciences, Anglia Ruskin University, Cambridge, CB1 1PT, UK; b The Queen Elizabeth Hospital Foundation Trust, King's Lynn, UK; c Institute of Mental Health Sciences, School of Health Sciences, Ulster University, Newtownabbey, BT37 0QB, UK; d Faculty of Science and Technology, Anglia Ruskin University, Cambridge, CB1 1PT, UK; e Cambridge University Hospitals NHS Foundation Trust, Cambridge, UK; f Vision and Hearing Sciences Research Center, Anglia Ruskin University, Cambridge, CB1 1PT, UK; g Pennine Acute Hospitals, NHS Trust, UK; h Department of Cancer Epidemiology and Prevention, Cancer Care Alberta, Alberta Health Services,Calgary, AB T2S 3C3, Canada

**Keywords:** Bladder cancer, Home-based exercise, Cancer survivor, Co-design intervention

## Abstract

**Background:**

Participation in physical activity has been found to be beneficial for mental and physical health outcomes among cancer survivors. However, to date no intervention exists specifically to promote physical activity among bladder cancer survivors. In light of this knowledge a home-based exercise intervention was co-created for those recently diagnosed with bladder cancer.

**Aim:**

The aim of the present study, financially supported by Action Bladder Cancer UK [1], is to pilot the home-based exercise intervention tailored specifically for bladder cancer survivors (i.e. from the point of diagnosis) to improve physical and mental health outcomes (during treatment and beyond) in this population.

**Methods:**

This study will use a randomised controlled trial design. Arm one will consists of the 14 week home-based exercise intervention and arm two usual care (15 participants will be randomised to each arm). Baseline data collection will take place shortly after clinical diagnosis of bladder cancer, and follow-up approximately 7 weeks and then again approximately 14 weeks after commencement of the intervention. At each data collection point data will be collected from participants relating to demographics, physical and mental health. Participants will aslo be asked to wear an Actigraph Accelerometer at each data collection point for seven consecutive days. Immediately after baseline data collection participants in the intervention arm will be given the home-based exercise booklet.

**Ethics and dissemination:**

Ethical approval was obtained for the present study via The London- City and East Research Ethics Committee (ID:291676). Results of this study will be disseminated through peer-reviewed publications and scientific presentations.

## 1 Introduction

Approximately, 10,000 people are diagnosed with bladder cancer every year and it's the tenth most common cancer in the UK. The condition is more common in older adults, with most new cases diagnosed in people aged 60 and above [2]. Importantly, the survival rate for bladder cancer (when compared to some other cancer types) is good. The general 5-year survival rate for people with bladder cancer is 77%, the overall 10-year survival rate is 70% and the overall 15-year survival rate is 65% [3].

There are many common physical and psychological consequences of cancer and treatment, including fatigue, pain, sleep disturbance, lymphoedema, weight gain, loss of muscle mass, cancer-related distress (e.g. fear of recurrence, financial concerns), anxiety, and depression [4,5]. Furthermore, the majority of people living with and beyond cancer are living with at least one other long-term chronic condition (e.g. hypertension, obesity, mental health conditions) [6]. Treatment for bladder cancer often includes surgery (including Transurethral bladder tumour resection, Radical cystectomy and lymph node dissection, Urinary diversion [stoma]), but may also include chemotherapy, immunotherapy, and targeted therapy. These treatments result in specific unwanted side effects including for example, mild bleeding and discomfort after surgery, erectile dysfunction, loss of sexual feeling and orgasm, frequent urination, and those with advanced bladder cancer may have been fitted with a uro-stoma [7].

With a high bladder cancer survival rate and thus a relatively large proportion of people living with and beyond bladder cancer [8] it is important to consider health behaviour interventions to improve general health, bladder cancer-specific complications, and general quality of life. One such intervention may include the promotion of physical activity. Indeed, physical activity has been shown to reduce cancer-related fatigue, pain, maintain an adequate weight and muscle mass, reduce levels of distress, anxiety and depression, reduce the risk of erectile dysfunction, and aid in the prevention and maintenance of several other non-communicable diseases [[9], [10], [11]]. In light of this knowledge The Independent Cancer Taskforce recommended that everyone diagnosed with cancer in the UK should have access to the elements of the Recovery Package by 2020. The Recovery Package is a series of key interventions which, when delivered together, can greatly improve outcomes for people living with and beyond cancer. The package includes an holistic needs assessment, treatment summary, cancer care review, health and wellbeing clinics. These elements are part of an overall support of self-management including physical activity as part of a healthy lifestyle, managing consequences of treatment, and information, financial and work support [12]. This was upheld in the recently published NHS Long Term Plan [13]. Physical activity, is highlighted in the Recovery Package due to evidence from randomized controlled trials (RCTs) of physical activity interventions that show improvements in fatigue, pain, sleep, lymphoedema, anxiety and depression, body composition and quality of life in people living with and beyond cancer [14]. Furthermore, there is a large body of observational evidence that shows that people living with and beyond cancer who are more active have reduced all-cause and cancer-specific mortality risk and a reduced risk of cancer recurrence [15].

However, despite the guidance levels of physical activity in cancer survivors are low. For example, in one study of US prostate cancer survivors objectively measured moderate-to-vigorous physical activity was low at all 4 time points (baseline, 5 week, 6 months and 12 months; median, 0.0–5.2 min/day), with no overall change across study assessments (global P = .29) [16]. Other studies in those living with and beyond other cancers have found similarly low levels of physical activity [17]. Therefore, interventions are urgently required to promote sustainable physical activity to those living with and beyond cancer. Moreover, to our knowledge to date no physical activity intervention has been developed and implemented specifically for those living with and beyond bladder cancer. Indeed, symptoms present in bladder cancer (differentiating from other solid tumour cancers) may further discourage individuals from participation in physical activity. Specifically, frequent urination and pain. Moreover, patients with advanced bladder cancer might also have a uro-stoma that can interfere with the perceived body image.^7^

## 2 Aim

The aim of the present study, financially supported by Action Bladder Cancer UK^1^, is to pilot a home-based exercise intervention tailored specifically for bladder cancer survivors (i.e. from the point of diagnosis) to improve physical and mental health outcomes (during treatment and beyond) in this population.

### 2.1 Setting

King's Lynn is a hanseatic town on the coast of Norfolk in England. It has a population of approximately 49,000 residents of whom approximately 50% are male and 59% are aged between 18 and 64 years with 18% aged over 65 years. Moreover, approximately 95% of the population are white British [18]. The CADENCE Trial will be carried out initially in The Queen Elizabeth Hospital King's Lynn Foundation Trust NHS (QEHKL). Participants will be recruited from one oncology clinic at the hospital where patients are under the care of one urological consultant (co-principal investigator). The patients will be recruited from the patients with newly diagnosed bladder cancer or diagnosed with recurrence of bladder cancer at the QEHKL. They will subsequently be booked for a transurethral resection of the bladder cancer. About 20–30% of these patients will need further treatment in the form of radical cystectomy.

## 3 Method and analyses

The evaluation of this pilot study will use a randomised controlled trial design. Baseline data collection will take place shortly after clinical diagnosis of bladder cancer. The duration of the entire study will be 4–5 months. Participants will be randomised by a trial coordinator (using simple random sampling) as they enter the study to either the control (usual care) or the intervention group (exercise) at baseline and prior to consent. Concealed allocation will take place. The unit of randomisation will be the participant.

### 3.1 Recruitment

Participants will be patients attending one urological consultant's oncology clinic or a flexible cystoscopy outpatient appointment and consecutively screened. If the patient meets the inclusion criteria the research team will provide them with a Participant Information Sheet and a Physical Activity Readiness Questionnaire at the point of clinical diagnosis. Approximately one week later the patient will be contacted by phone by either a trained research assistant or a member of the urological consultants care team. A one week window will be given to allow the patient time to read study material. The patient will be asked if they would like to take part in the study and if the Physical Activity Readiness Questionnaire deemed it okay for them to exercise (if exercise is not deemed acceptable the participant will not be recruited into the study). If the patient agrees to take part, an appointment will be made for them at QEHKL for consent and baseline data collection. Once recruited, patients will be randomised to usual care or exercise intervention. On arrival, for baseline data collection, and before any data is collected participants will be asked to provide written informed consent. Participants will be asked if they have any questions and will be told they can withdraw from the study at any point without giving reason and no adverse action will be taken as a result of this.

### 3.2 Participants inclusion/exclusion criteria

The study will be restricted to adults (age ≥18) who are able to consent for themselves (presumed from completion of the consent form) and individuals who receive a diagnosis of non-metastatic (stage I–III) bladder cancer. Patients will also be screened using the Physical Activity Readiness Questionnaire. Patients will be excluded if they are unable to provide informed consent due to severe cognitive impairment, and have metastatic disease (stage IV).

### 3.3 Intervention

A self-guided printed home-based exercise booklet has been co-created for those with bladder cancer (see supplementary material). The content allows patients to achieve the World Cancer Research Foundations physical activity guidelines [1] being active every day for 30 min or more (and aiming for 10,000 steps a day) [2], getting stronger and fitter (doing strength, balance and stretching exercise twice per week and two 30 min sessions of vigorous exercise per week) [19]. The booklet was co-created through an iterative process. First, the booklet was developed and designed by a sport scientist and a urological surgeon. Next, the content of the booklet was shared with patients, health professionals (urologists, oncology physiotherapist, and personal trainers) and experts (academics) in the field, who were asked to comment on the appropriateness of the content and make suggestions for improvement. The sport scientist and the urological surgeon then utilised this feedback to improve the booklet. This process continued until all involved were happy with its content.

### 3.4 Control group

All participants in the control group will complete all evaluation measures at each time point. Control participants will also be required to complete informed consent prior to collecting any measurements.

### 3.5 Evaluation

All evaluation measurements will be taken at baseline, approximately 7 weeks and then again approximately 14 weeks after commencement of intervention (we say approximately as the timing depends on when the patients scheduled appoints occur).

### 3.6 Measures

After randomisation a trained research assistant will collect baseline data on participants demographic data including age, sex, education, current employment status, marital status and living arrangement (who they live with), and ethnicity by way of survey. Participants’ waist circumference (WC), height and weight (to calculate body mass index) will also be recorded. As well as body fat percentage and muscle mass using bio-impedance scales. Handgrip strength will be recorded using the handheld dynamometer. Fatigue will be assessed using the 13-item fatigue subscale of the Functional Assessment of Chronic Illness Therapy-Fatigue (FACIT-F) questionnaire [20]. Sleep will be assessed using the Pittsburgh Sleep Quality Index [21], an 18-item questionnaire which assesses sleep quality and disturbances over a 1-month time interval. Quality of life will be assessed using the five-level EuroQol-5D questionnaire (EQ-5D-5L) [22]. Finally, participants will be fitted with the Actigraph accelerometer to measure free-living physical activity over 7 consecutive days. Follow-up data will be collected at 7 weeks and 14 weeks.

Patients randomised to the control comparator will receive ‘usual care’ and undergo all baseline and follow-up measures.

Levels of free-living physical activity will be assessed using the Actigraph accelerometer (GT3X) at each data collection point. The Actigraph accelerometer is a valid and reliable tool to monitor free-living physical activity and its validity and reliability has been shown in multiple populations [23]. The Actigraph GT3X is worn on a belt around the waist with the device itself positioned above the right hip either over or under clothing. We will employ a sampling frequency of 30 Hz. Service users will be asked to wear the device during waking hours every day for seven consecutive days, but not during water-based activities or sleep.

In addition to the above we will also collect data on the following variables: number of patients screened, proportion of patients enrolling, and number of patients who dropout of the study.

Those in the intervention arm will also be asked to take part in one semi-structured qualitative interview. Interviews will explore experiences of the intervention including barriers and facilitators to compliance, satisfaction and perceived effectiveness of the different elements of the intervention, and suggestions for improvement. All interviews will be audio recorded and transcribed. With the consent of participants, semi-structured interviews will be conducted. We anticipate that each interview will take approximately 30–45 min. Interviews will be digitally recorded, and transcripts will be anonymized for identifiable information.

### 3.7 Analysis

#### 3.7.1 Outcome

The primary outcome for this study will be indicative change in average daily time spent in sedentary time, light physical activity and moderate-to-vigorous physical activity (MVPA) as recorded by the Actigraph accelerometer. Other secondary outcomes collected using participant questionnaires and objective measures include: (1) indicative change in WC and BMI, (2) indicative change in fatigue, sleep and QOL, (3) indicative change in grip strength.

### 3.8 Sample size

The aim of the present study is to pilot the proposed intervention and if promise is observed we plan to secure further funding to incorporate the intervention booklet into a mobile application that we will subsequently run a fully powered RCT. As in this scenario we are predominantly interested in “promise” we will recruit and trial the intervention in a sample of 30 participants (15 in the control arm and 15 in the intervention arm). It should be noted here that findings from this pilot will be used to inform the power calculation for the full trial.

### 3.9 Data analysis-Quantitative

We will summarize all data by treatment group. Statistical measures of central tendency and dispersion such as mean, standard deviation (continuous variables), frequency distribution, interquartile range (IQR) and median (categorical variables) will be used in descriptive statistics. Non-parametric statistical methods will be used for bivariate analysis. Stata version 16 software will be used to perform the analysis.

### 3.10 Data analysis-qualitative

Content analysis will be the first analysis method used but where possible other thematic analysis such as Framework analysis or Interpretative Phenomenological Analysis may also be conducted to fit study objectives. QSR Nvivo software will be used for data management.

### 3.11 Patient and public involvement

The exercise intervention was developed through an iterative process and consultation with input from patients, health professionals (urologists, oncology physiotherapist, personal trainers) and experts (academics) in the field. Moreover, this study will interview patients on study exit to further precise the intervention.

### 3.12 Ethical considerations and dissemination

Explicit written informed consent will be sought from all study participants. All participants will be informed that they have the right to withdraw from the programme at any point without giving reason.

The results of this evaluation will be disseminated to academic audiences through presentations at national and international conferences in physical activity, public health and medicine and through peer-reviewed publications in relevant journals. Results will be disseminated to the public, policy makers, and other charities through seminars and press releases co-ordinated through the Anglia Ruskin University Press Office.

Ethical approval was obtained for the present study via The London- City and East Research Ethics Committee (ID:291676).

## 4 Discussion

The present protocol is to pilot a home-based exercise intervention co-created through an iterative process and tailored specifically for bladder cancer survivors to improve physical and mental health outcomes in this population. If the intervention shows promise we will subsequently incorporate the intervention booklet into a mobile application and run a fully powered RCT.

A clear strength of this study will be the utilisation of a home-based exercise intervention tailored specifically for bladder cancer survivors to promote physical activity and implementing the intervention from the point of diagnosis (approximately one week after). However, some limitations may exist. First, owing to the “high-risk” population we may experience a high level of drop out or a low uptake. It is possible that as someone goes through bladder cancer treatment taking up physical activity may not be a priority for them. Second, the Actigraph GT3X are calibrated to record ambulatory activity (hip movement) and therefore underestimate the intensity of some activities. Third, bladder cancer is most common among older adults who also have a high prevalence of multiple non-communicable diseases which may further impact their ability to engage in a home-based exercise program.[24].

## Declaration of competing interest

The authors declare that they have no known competing financial interests or personal relationships that could have appeared to influence the work reported in this paper.
