# Supplementary material for: The CADENCE pilot trial – Promoting physical activity in bladder cancer survivors: A protocol paper
Source: Contemp Clin Trials Commun. 2021 Jun 18;22:100809. doi: 10.1016/j.conctc.2021.100809 (PMC8239430; doi:10.1016/j.conctc.2021.100809)
Supplement: Multimedia component 1 [file mmc1.pdf]

THE CADENCE TRIAL

Physi**ca**l activity in blad**de**r ca**nce**r

The Cadence Trial is being run to assess the benefits of regular physical activity in people diagnosed with bladder cancer.

With your help, we are aiming to develop an activity plan that we can use to improve both the quality, and the quantity, of life.

What are the benefits of physical activity?

Research on physical activity among people with cancer has shown the following benefits:

- Increased energy levels
- Relief from pain and lymphoedema
- Improved sleep quality and body composition
- Reduced anxiety and depression
- Enhanced quality of life

What is the activity plan?

Because of this evidence, this activity plan has been designed by oncologists, GPs, physiotherapists, and scientists to help you achieve the levels of physical activity recommended by the Independent Cancer Taskforce:

- **Aerobic activities** for a total of **150 minutes per week** performed in bouts/sessions/rides of at least 10 minutes across **five days per week**.
- **Strength activities** involving major muscle groups (like chest or upper legs) on **more than two days per week**.
- **Breath work** in addition to the other activities, **5 minutes each day.**
- **Note:**
    - **Balance training incorporated** into aerobic/strengthening activities.
    - **Rest when you need to rest**, only you know how hard you are pushing.

What level of activity is required?

Moderate intensity activities require some effort and make you breathe harder than normal, whilst still being able to hold a conversation. Examples include:

- Walking or cycling on a flat surface at relaxed pace
- Leisurely swimming
- Playing tennis in doubles
- Ballroom dancing
- Table tennis and bowling
- Yoga
- Mowing, sweeping and gardening
- Painting and decorating
- Washing windows
- Cleaning gutters
- Caring for loved ones (washing and dressing etc)

Example plan

Day	Activity	Total minutes
**Monday**	**Aerobic**<br>Do the following 2 times, with 1 minute rest in between each circuit:<br>● High knees for 1 minute<br>● Marching on the spot for 2 minutes<br>● Jumping Jacks for 1 minute<br>● Side stepping for 2 minutes<br>● Rest for 1 minute<br>**Strength upper body**<br>Do the following 4 times, with 1 minute rest in between each circuit:<br>● Wall pushes 10 times<br>● Banded rows 10 times<br>● Arm raises 10 times<br>● Arm curls 10 times<br>● Bridge 10 seconds	14 minutes aerobic<br>20 minutes strength
**Tuesday**	**Aerobic**<br>Do the following 4 times, with 1 minute rest in between each circuit:<br>● High knees for 1 minute<br>● Marching on the spot for 2 minutes<br>● Jumping Jacks for 1 minute<br>● Side stepping for 2 minutes<br>● Rest for 1 minute	28 minutes aerobic
**Wednesday**	Rest	
**Thursday**	**Aerobic**<br>Do the following 2 times, with 1 minute rest in between each circuit:<br>● High knees for 1 minute<br>● Marching on the spot for 2 minutes<br>● Jumping Jacks for 1 minute<br>● Side stepping for 2 minutes<br>● Rest for 1 minute<br>**Strength lower body**<br>Do the following 4 times, with 1 minute rest in between each circuit:<br>● Sit to stand 10 times<br>● Squats 10 times<br>● Heel raises 10 times<br>● Sideways leg lifts 10 times<br>● Hip extensions 10 times	14 minutes aerobic<br>20 minutes strength
**Friday**	**Aerobic**<br>Do the following 4 times, with 1 minute rest in between each circuit:<br>● High knees for 1 minute<br>● Marching on the spot for 2 minutes<br>● Jumping Jacks for 1 minute<br>● Side stepping for 2 minutes<br>● Rest for 1 minute	28 minutes aerobic
**Saturday**	Rest	
**Sunday**	**Aerobic**<br>Do the following 4 times, with 1 minute rest in between each circuit:<br>● High knees for 1 minute<br>● Marching on the spot for 2 minutes<br>● Jumping Jacks for 1 minute<br>● Side stepping for 2 minutes<br>● Rest for 1 minute	28 minutes aerobic

Aerobic activities - Instructions

Marching on the spot - seated

1. Lift your feet up as if you are marching on the spot
2. Repeat movements for 30 seconds, then rest for 30 seconds
3. Repeat three times
4. Rest for 1-2 minutes before next activity

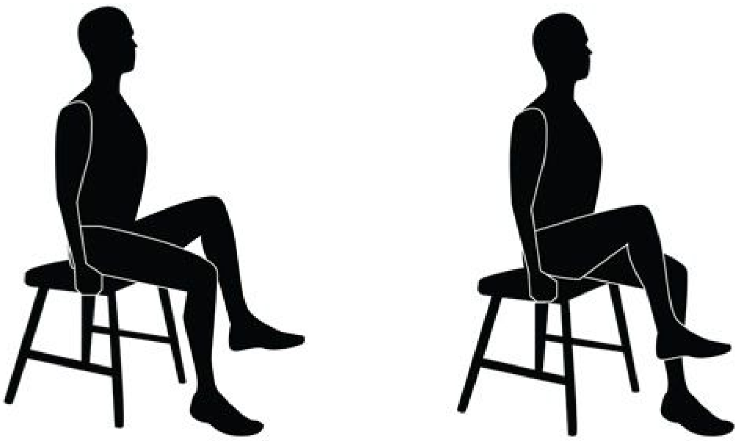

Marching on the spot - standing

1. March on the spot (hold onto something if you need to)
2. Repeat movements for 30 seconds, then rest for 30 seconds
3. Repeat three times
4. Rest for 1-2 minutes before next activity

   **Do not do this if you feel dizzy or unsteady

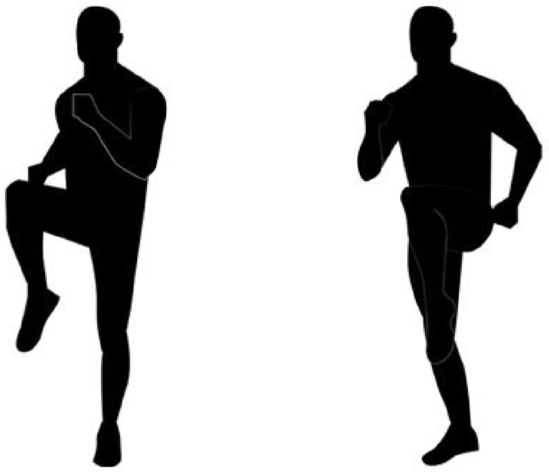

Jumping Jacks - seated

1. Start with your arms raised above as high as you can
2. Bring your arms down to the side in a big arc
3. Repeat movements for 30 seconds, then rest for 30 seconds
4. Repeat six times
5. Rest for 1-2 minutes before next activity

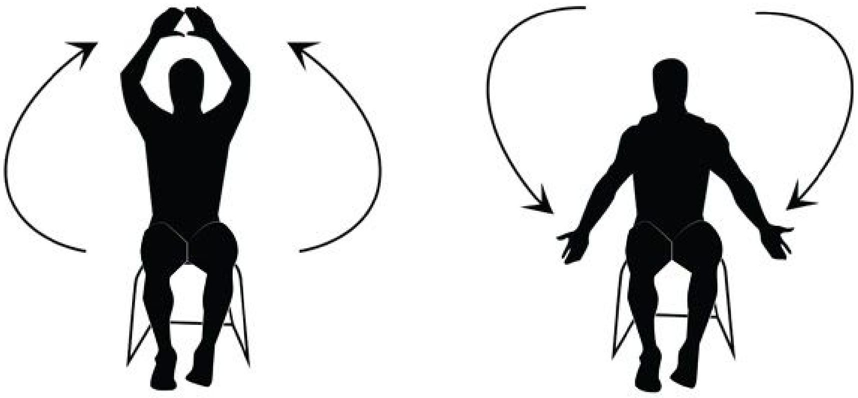

Jumping Jacks - standing

1. Step sideways with your right foot, by about a foot, keeping your left foot still
2. At the same time raise your right arm out to the side
3. Return to the start
4. Repeat on the left side, keeping your right foot still
5. Repeat movements for 30 seconds, then rest for 30 seconds
6. Repeat three times
7. Rest for 1-2 minutes before next activity

   **Do not do this if you feel dizzy or unsteady

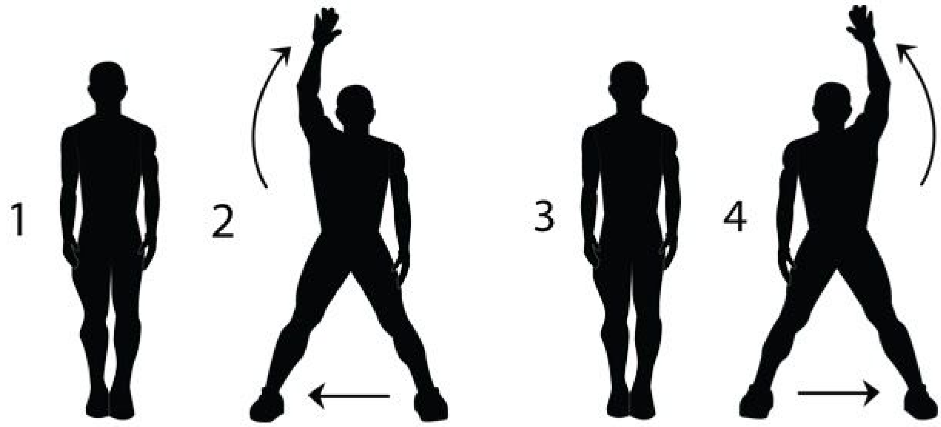

Elbow side steps

1. Bring elbows together in front of you and bend at 90 degrees with your palms together
2. Side step to right and open your arms to the sides, with your elbows bent at 90 degrees
3. Return to the start
4. Repeat to left side, keeping your right foot still
5. Repeat movements for 30 seconds, then rest for 30 seconds
6. Repeat three times
7. Rest for 1-2 minutes before next activity

   **Do not do this if you feel dizzy or unsteady

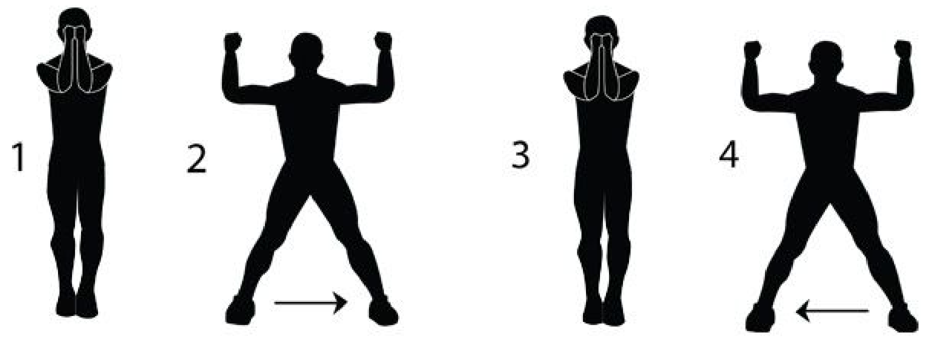

Strength activities

Wall pushes

1. Stand at arms-length from a wall, place both hands on the wall at chest height, fingers upwards
2. Lean forward by bending at your hips and elbows
3. Do this carefully so that you feel safe and supported throughout
4. Push the wall away from you, by straightening your arms and hips
5. Do this movement 10 times, then rest for 30 seconds
6. Repeat three times
7. Rest for 1-2 minutes before next activity

   **Do not do this if you feel dizzy or unsteady

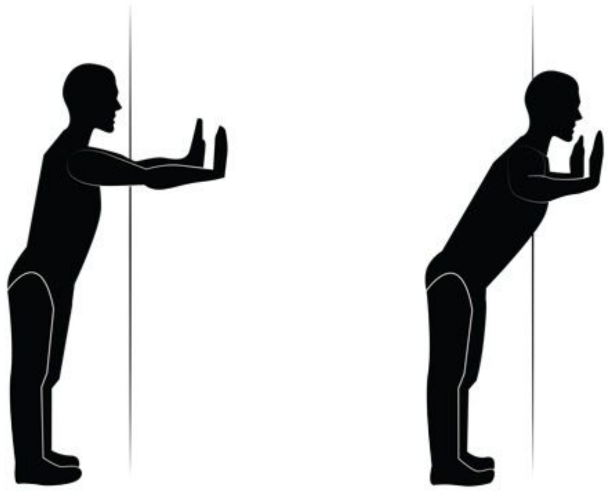

Banded row

1. Sit, or stand, depending on how you feel. Make sure you are safe and secure
2. Attach one end of a band to a wall or door handle
3. Hold the other end of the band and pull, imagine trying to pull your elbows by your ears
4. You should feel your shoulder blades come together
5. Hold for 2 seconds, counting out loud, then slowly return to starting point
6. Do this movement 10 times, then rest for 30 seconds
7. Repeat three times
8. Rest for 1-2 minutes before next activity

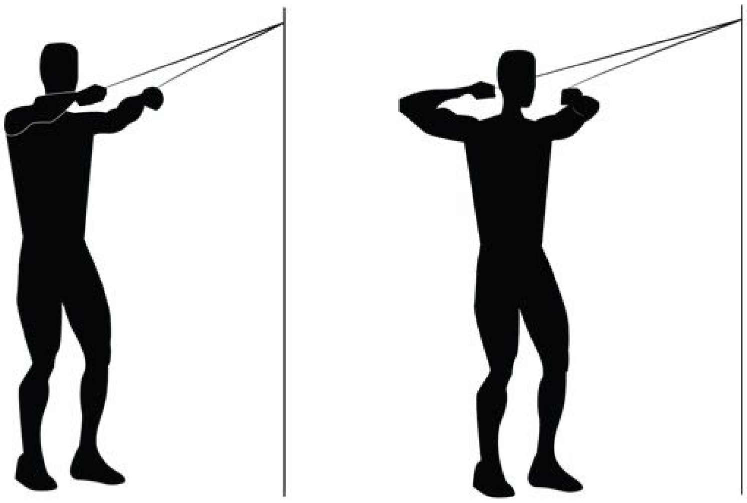

Arm lifts

1. Sit, or stand, depending on how you feel. Make sure you are safe and secure
2. Keep elbow straight, lift your arms out to the sides as far and high as you can
3. Aim to make a T-shape with your body and your arms - imagine you're a scarecrow
4. Hold for 2 seconds, counting out loud
5. Slowly lower your hands to the starting point, with your hands by your sides
6. Do this movement 10 times, then rest for 30 seconds
7. Repeat three times
8. Rest for 1-2 minutes before next activity
9. If you can't do this with both hands at once, try one hand at a time

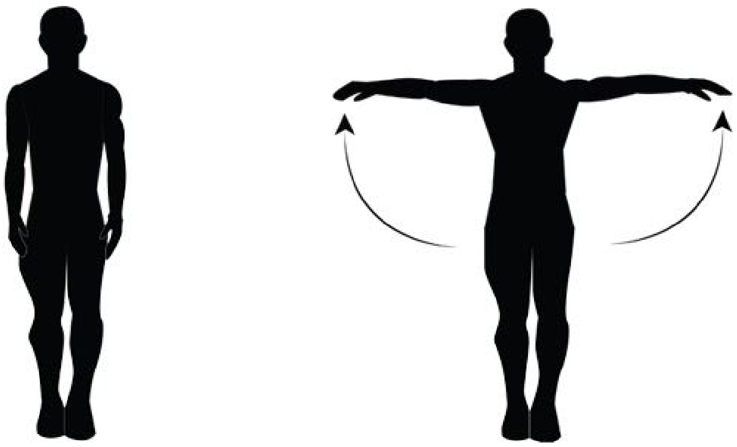

Arm curls

1. Sit, or stand, depending on how you feel. Make sure you are safe and secure
2. Hold something in each hand, such as two tins of beans, with your hands at your sides
3. Bend at elbow curling your tins of beans up to the front of your shoulders
4. Slowly lower to starting point
5. Do this movement 10 times, then rest for 30 seconds
6. Repeat three times
7. Rest for 1-2 minutes before next activity
8. If you can't do this with both hands at once, try one hand at a time

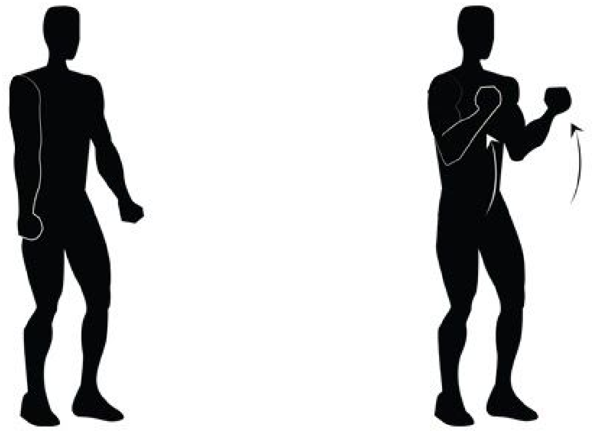

Bridge

1. Lay down on your back, arms by your side, palms facing down
2. Bend knees so that your feet are flat on the floor
3. Press your feet into floor and lift bum up off the floor
4. Hold for 2 seconds, counting out loud
5. Slowly lower your bum back down to the floor
6. Do this movement 10 times, then rest for 30 seconds
7. Repeat three times
8. Rest for 1-2 minutes before next activity

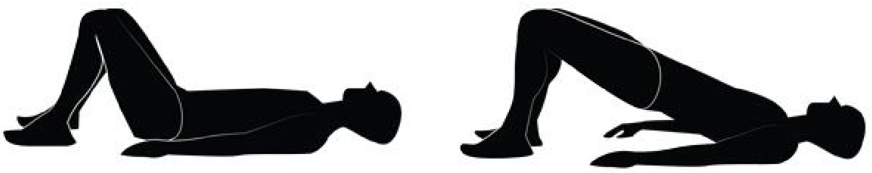

Sit to stand

1. Sit on the edge of chair, feet hip-width apart, holding onto the chair arms if you need
2. Lean slightly forwards
3. Stand up slowly pushing off on your legs, not your arms
4. Make sure you look forward not downwards
5. Stand upright and pause, then slowly sit down
6. Do this movement 10 times, then rest for 30 seconds
7. Repeat three times
8. Rest for 1-2 minutes before next activity

   **Do not do this if you feel dizzy or unsteady

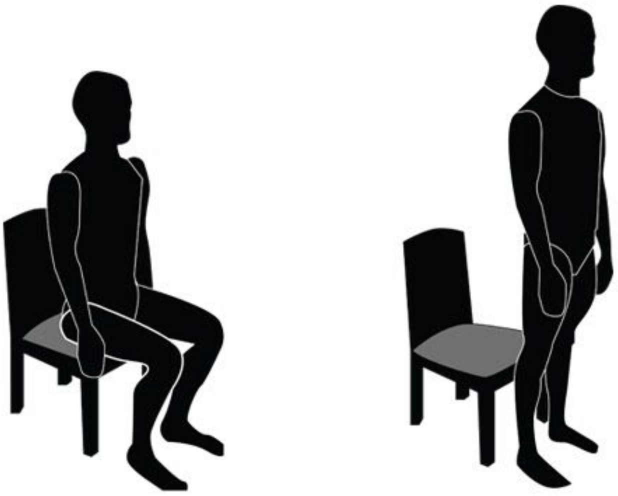

Squats

1. Stand holding onto something firm, such as a heavy chair
2. Slowly squat down, bending your knees as far as is comfortable
3. Keep your back straight
4. Gently come up to upright, squeezing your buttocks as you do so
5. Do this movement 10 times, then rest for 30 seconds
6. Repeat three times
7. Rest for 1-2 minutes before next activity

   **Do not do this if you feel dizzy or unsteady

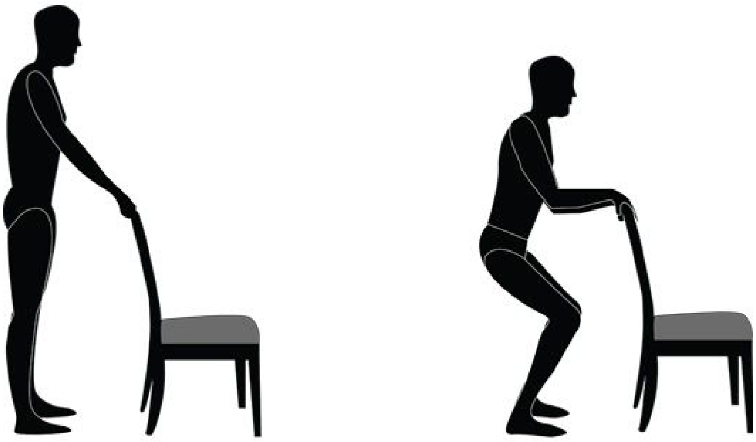

Heel raise

1. Stand holding onto something firm, such as a heavy chair
2. Slowly lift both heels off the floor as far as comfortable, standing on your tiptoes
3. Keep your back straight
4. Slowly return to starting point
5. Do this movement 10 times, then rest for 30 seconds
6. Repeat three times
7. Rest for 1-2 minutes before next activity

   **Do not do this if you feel dizzy or unsteady

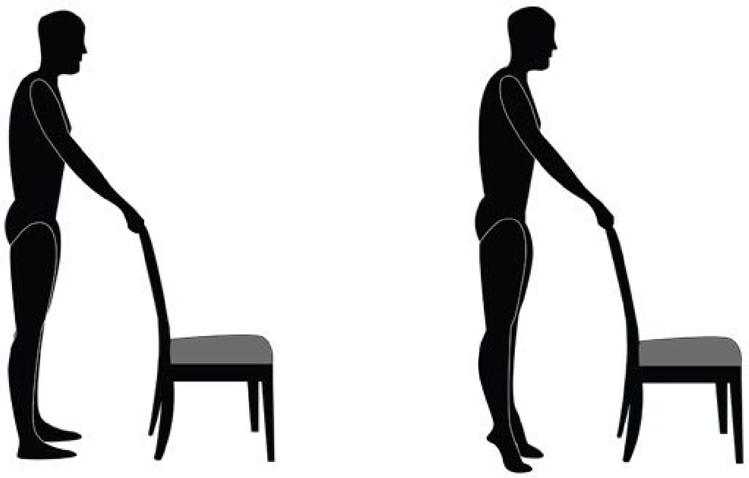

Sideways leg raise

1. Stand holding onto something firm, such as a heavy chair
2. Raise your right leg out to the side, keeping your left foot on the floor
3. Keep your back and hips straight
4. Slowly return to starting point
5. Do this movement 10 times, then rest for 30 seconds
6. Repeat three times
7. Rest for 1-2 minutes before next activity

   **Do not do this if you feel dizzy or unsteady

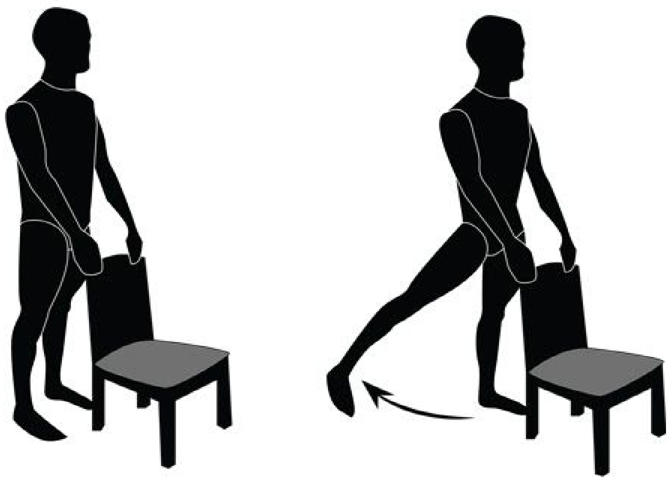

Hip extension

1. Stand holding onto something firm, such as a heavy chair
2. Raise right leg backwards as far as comfortable, keeping your left foot on the floor
3. Keep your back straight
4. Slowly return to starting point
5. Repeat with left leg
6. Do this movement 10 times, then rest for 30 seconds
7. Repeat three times
8. Rest for 1-2 minutes before next activity

   **Do not do this if you feel dizzy or unsteady

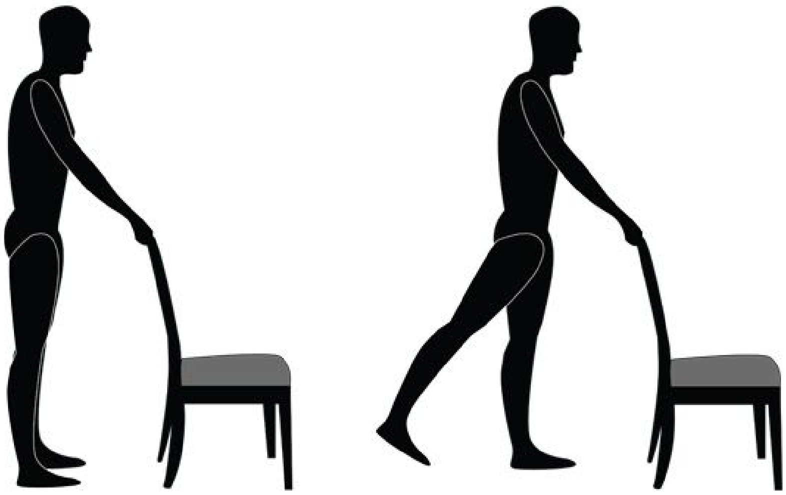

Breathing activities

In these activities you will focus on the way in which you breath for anything between 5 minutes and 1 hour each day. This is in addition to the aerobic and strength activities. You can choose how much time you can, and need to, spend on breathing activities. Some people enjoy doing it at the start of the day to give them energy and a positive approach to the day, other people prefer to do it at the end of the day to help them relax and unwind.

Benefits of breathing activities

The benefits of controlling the way in which we breathe has been explored through activities such as yoga, meditation and mindfulness. These benefits can be split into physical and psychological:

**Physical**	**Psychological**
Improved blood flow	Reduced anxiety and stress
Stimulation of lymphatic flow	Reduced feelings of depression
Improved digestion	Improved sleep
Increased energy levels	Improved mindfulness

What is mindfulness?

Mindfulness is an enhanced awareness of your physical and mental state. This awareness allows you to take control over some of your thoughts. This control can be used to better understand what you are thinking, and why; it can also be used to clear some of those thoughts that get stuck in our head when we are worried or anxious. With this understanding of ourselves, and with clearer minds, we sleep better and as a result have more energy.

Apps for mindfulness and anxiety

The NHS has a list of useful apps that help with mental health challenges, these include apps for mindfulness, anxiety, stress and depression. The Mindfulness apps will help with your breathing techniques.

These can be found on the NHS website: www.nhs.uk/apps-library/category/mental-health/
Or, use Google to search for: “NHS Mental Health Apps”

Breathing activity instructions

Belly breathing

1) Sit or lay down in a comfortable position.
2) Place your left hand on your chest, just over your heart and your right hand on your belly just below your ribs.
3) Keeping your lips lightly closed, take a deep breath in through your nose by using your belly, trying not to move your chest. Your right hand (on your belly) should move up, whilst your left hand (on your chest) should stay still.
4) As you exhale, purse your lips together (imagine you are whistling) and allow the belly to sink in and feel your right hand on your belly move down as you do this. At the end of the breath you'll feel your belly go down and then back up a little.
5) Take your time with this, doing it slowly is the only way it will work.
6) There is no time limit for this.

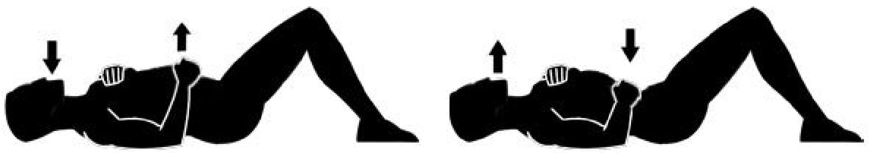

4, 7, 8 breathing

This activity is best lying down, however, you can do it sitting if you are not able to. This is a really relaxing breathing technique and one to do if you are ever having trouble sleeping.

1) Place your left hand on your chest, just over your heart and your right hand on your belly just below your ribs.
2) Put your tongue to the roof of your mouth, just behind your teeth and inhale through your nose for a count of **four**.
3) When you feel your lungs are full, hold your breath and count to **seven** in your head.
4) Then slowly exhale through your nose making it last whilst you count to **eight** in your head. When you reach eight your lungs should be empty.
5) Repeat as many times as you need to in order to find a feeling of calm.

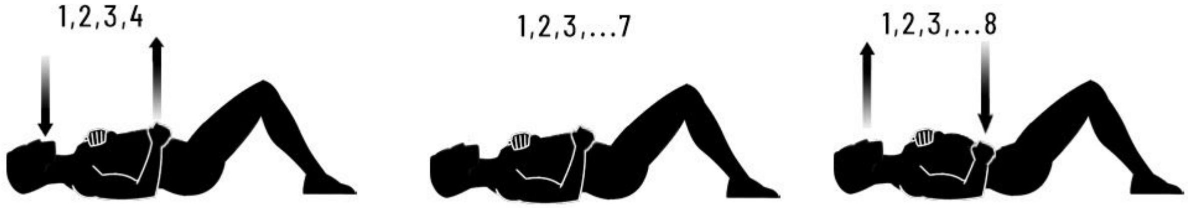

For more information about this trial, or about these exercises, please contact:

Dr Lee Smith
lee.smith@aru.ac.uk
